# Revisit to the mechanism of quenching: Power effects for sonochemical reactions^☆^

**DOI:** 10.1016/j.ultsonch.2025.107419

**Published:** 2025-06-06

**Authors:** Ryota Aoki, Kanji D. Hattori, Takuya Yamamoto

**Affiliations:** Department of Chemical Engineering, Graduate School of Engineering, Osaka Metropolitan University, 1-1, Gakuen-cho, Naka-ku, Sakai, Osaka 599-8531, Japan

**Keywords:** Ultrasound, Sonochemistry, Quenching, Wave superposition, Acoustic cavitation

## Abstract

In this study, the mechanism that the sonochemical reactions are quenched due to an increase in ultrasonic power was investigated through six experiments, stability analysis, and numerical simulations. The experiments involved measuring the sonochemical reaction rate, observing sono-chemiluminescence (SCL), conducting particle image velocimetry (PIV) measurement, measuring sound pressure, observing bubble motion, and measuring the degassing rate of dissolved oxygen. Through these experiments and numerical simulations, the phenomena could be classified into three regions in response to ultrasonic power. In the region of small ultrasonic power, the superposition of ultrasound is good, and the reaction rate increases with the ultrasonic power. However, at higher ultrasonic power, the superposition of ultrasound is deteriorated, suppressing the bubble nucleation and growth due to rectified diffusion. This results in a lower fluid flow velocity due to acoustic streaming, a smaller reaction rate, and smaller degassing rate. At much higher ultrasonic power, the ultrasonic standing waves are changed into traveling waves resulting in bubble cluster formation and movement, as well as a smaller chemical reaction rate. These experimental results and the proposed mechanisms were also supported by the numerical simulation and stability analysis results.

## 1 Introduction

When ultrasound is irradiated into an aqueous solution, it propagates through the solution, while acoustic cavitation occurs when high-power ultrasound is irradiated [1]. Bubble nuclei form when the local pressure becomes much smaller than vapor pressure due to ultrasound [1,2]. The nucleated bubbles grow due to bubble coalescence and rectified diffusion [1,3]. When the local pressure decreases due to ultrasonic oscillations, the bubble expands and vice versa. However, the largely grown bubbles continue to expand due to the fluid inertia around the bubble. The largely expanded bubbles are rapidly compressed, and the inner temperature and pressure instantaneously become larger than 5000 K and 100 atm., respectively. At the moment of bubble's compression, the molecules in the bubble are thermally decomposed, and radicals are generated [4,5]. The radicals react with the chemical species near the bubble wall in the aqueous solution. Various chemical reactions proceed from this radical formation and reaction. In addition to this chemical effect, many physical phenomena occur simultaneously. For instance, a micro jet is formed under the condition where an ultrasound field becomes asymmetric [[6], [7], [8], [9]], and capillary waves form at the gas–liquid interface. These chemical and physical effects can be used for many applications such as emulsification [[10], [11], [12], [13]], atomization [14,15], organic chemical decomposition [[16], [17], [18], [19]], cleaning [[20], [21], [22]] and etc.

The chemical reaction occurring during ultrasonic irradiation is called sonochemical reaction. This reaction is unique because the chemical reaction is initiated by the physical phenomena explained above. Hence, this reaction has been investigated by many researchers so far. The chemical reactions in the acoustic cavitation have been investigated for a single bubble environment [2,[23], [24], [25]]. In this system, the direct comparison between experimental and numerical results is possible [26]. Therefore, the phenomena in a single bubble have been experimentally measured and numerically modeled [27]. Meanwhile, the sonochemical reactions in a multi-bubble environment are extremely complicated phenomena. For example, the propagation of ultrasound is changed due to bubble oscillations [[28], [29], [30], [31]], and the bubble clusters and bubble structure form causing change in the bubble oscillation [32,33]. In addition, the bubble–bubble interactions and shock waves emitted from the bubbles also change the bubble oscillation and related chemical reactions. However, it is almost impossible to observe all of these phenomena simultaneously. For these reasons, the relationship between the bubble oscillation and chemical reactions in a multi-bubble environment has been studied so far and unclear phenomena still remain. Among them, this study focuses on the quenching of sonochemical reaction in a multi-bubble environment.

The sonochemical reaction rate decreases drastically at high-power condition [[34], [35], [36], [37], [38]] and solute addition [[39], [40], [41], [42]], and this phenomenon is called quenching. The solute-induced quenching has been explained by the vapor pressure and solute adsorption at the bubble-solution interface, and the mechanism was clearly explained. Meanwhile, the mechanism of power-induced quenching has many unclear points. Hatanaka *et al.* [34,35] explained the mechanism of power-induced quenching in terms of bubble clustering, and later Tuziuti *et al.* and Lee *et al.* explained that the quenching is caused by the decrease in the ratio of standing waves and particles addition improves the quenching condition [[36], [37]]. Recently, Asakura and Yasuda [38] explained that the quenching occurs due to the suppression of bubble formation. However, the detailed experimental evidence about the suppression of bubble formation was not shown and the whole phenomena occurring during quenching have not been clarified.

In this study, to understand the whole phenomena and the detailed mechanism of power-induced quenching, we conducted six experiments, stability analysis, and numerical simulations. Through these experimental and numerical results, the detailed mechanism of power-induced quenching was integrally explained.

## 2 Experiment

**Fig. 1:**
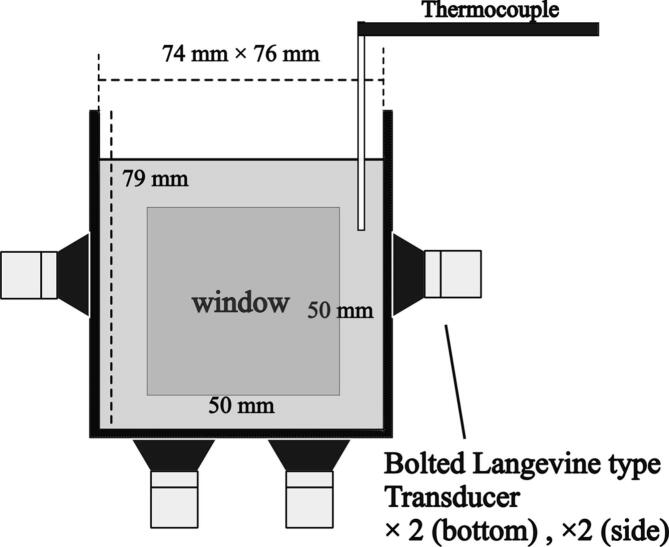
Experimental setup used in this study.

As shown in Fig. 1, an ultrasonic bath is rectangular made from stainless steel with two glass windows, the size of which is 50 and 50 mm. The dimensions of the vessel are 74 mm × 76 mm × 79 mm, and four transduces were fixed to the vessel walls: two transducers were attached to the bottom and two to the opposite sides. The bolted Langevin-type transducers were used, and the frequency of ultrasound was 154 kHz. The transducers were connected to the ultrasonic generator (Kaijo, QUAVA-multi). We filled the vessel with 400 mL of solution, and an acrylic plate was placed on the surface of liquid to avoid the effect of free surface motion on the sonochemical reactions.

In this study, six experiments were conducted to clarify the mechanism of quenching. The chemical reaction rate and the reaction efficiency were evaluated by potassium iodide (KI) dosimetry, and the reaction zone was evaluated by sonochemiluminescence (SCL) observation. In addition to the chemical reaction, we also measured the fluid flow velocity in the vessel using a particle image velocimetry (PIV) measurement. The ultrasonic pressure was measured by a hydrophone. The bubble motions were observed using a highspeed camera, and the degassing rate of dissolved oxygen was measured.

Before these experiments, the effective electric power was measured by a calorimetry method using a K-type thermocouple (Okazaki Manufacturing company, AEROPAK) and data logger (GRAPHTEC, midi LOGGER GL 220). During this experiment, the ultrasonic vessel was covered by a glass-fiber heat insulator to measure the generated heat accurately. The measured time variation of temperature in the vessel was converted into the ultrasonic power, *P*_US_ as follows.

(1) $$P_{US}=MC_{p}\frac{dT}{dt}$$

where *M* is the mass of pure water [kg], *C*_p_ is the heat capacity [J/(kgK)], *T* is the temperature [K], and *t* is time [s].

### 2.1 KI dosimetry

In the KI dosimetry, 400 mL of 0.10 mol/L KI solution was prepared, and air was injected into the solution for ten minutes to standardize the initial concentration of dissolved gas. Ultrasound was irradiated for 150 s, and 3 mL solution was sampled every 30 s. The acoustic cavitation creates OH radical and other oxides that create I^3-^ in the solution. By measuring the concentration of I^3-^, the chemical reaction rate was indirectly measured. The concentration of I^3-^ was measured using an ultraviolet–visible absorption spectrometer (SHIMADZU, UV-2600i). The chemical reaction rate and the sonochemical efficiency (SE) were calculated using the time variation of I^3-^ concentration. The SE evaluates the chemical reaction efficiency of ultrasonic, which is defined as

(2) $$SE=\frac{\mathrm{EV}}{P_{US}\varepsilon lt}$$

where *E* is the absorbance of I^3-^ [-], *V* is the volume of the KI solution [L], *ε* is the molar extinction coefficient of I^3-^ [L・mol^−1^・cm^−1^], *l* is the cuvette length [cm], *t* is irradiation time [s]. The values of *ε* and *l* were 26,303 L・mol^−1^・cm^−1^, and 1 cm, respectively. The reaction rate *k*_I3_ [mol・s^−1^] was calculate as

(3) $$k_{I3}=SE\times P_{US}$$

In this experiment, the temperature of the solution was maintained at 25 ± 5 ℃.

### 2.2 Sonochemiluminescence (SCL) using luminol aqueous

The chemical reaction reactivity and zone were measured by chemiluminescence of luminol solution. In this observation, the aqueous solution of 0.01 wt% luminol and 0.5 wt% sodium carbonate (Na_2_CO_3_) was used. The luminol in the solution reacts with hydroxyl radicals and other oxidants, and it emits blue light, which was captured using electron multiplying CCD camera (Andor, iXon Ultra, DC-888U3-CS0-EXF). Ten images were acquired at a frame rate of 5.0 fps and the exposure time was 0.10 s. The reaction intensity was evaluated by the luminescence intensity. These images were taken at 15 s after irradiation, when a stable ultrasonic field was formed.

### 2.3 Particle image velocimetry (PIV) measurement

The PIV measurement was conducted to measure the fluid velocity in the vessel in the same way as our previous study [43]. Fluorescent particles (Rhodamine B, EB-FLUOSTAR, KANOMAX, particle size: 15 μm) were dispersed in pure water, and a sheet of green laser was irradiated from above through the acrylic plate on the free surface using 3 W CW-YAG laser, the wavelength of which was 532 nm. The fluorescent particles were excited by the green laser sheet, and they emitted fluorescent light. The peak wavelength of fluorescence was about 580 nm. A high-pass filter, which cuts light below the wavelength of 570 nm, was used to capture only the fluorescent light from the particles. The particle motions were recorded by a high-speed camera (Photron, FASTCAM Mini AX100). The frame rate of high-speed camera was 250 fps. Totally, 5487 images were taken. Photographs were started to be taken at 15 s after the ultrasonic irradiation, when a stable ultrasonic field was formed. Finally, the recorded particle motion was converted to flow velocity using a digital image correlation method (OpenPIV, Python package).

### 2.4 Measurement of sound pressure

A local acoustic pressure in the vessel was measured using a hydrophone (Neptune Sonar, D/300 Miniature Hydrophones) with a frequency response band of 0–400 kHz. The electric signal from the hydrophone was measured by an oscilloscope (Tektronix, TBS2202B). In this experiment, an acrylic plate with a hole in the center was placed on the liquid surface, and the hydrophone was immersed into the water through the hole. The hydrophone was placed at the center of the ultrasonic bath in the horizontal direction, and the distance between the bottom of the vessel and the tip of the hydrophone was set at 31 mm. The sound pressure was measured at 15 s after irradiation, when a stable ultrasonic field was formed.

**Fig. 2:**
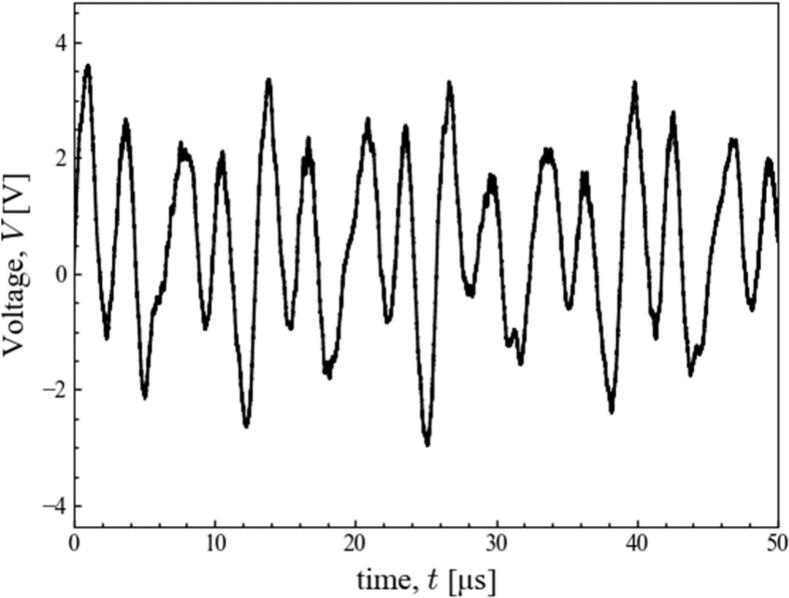
Time variation of voltage measured by the hydrophone at the ultrasonic power of 69 W.

**Fig. 3:**
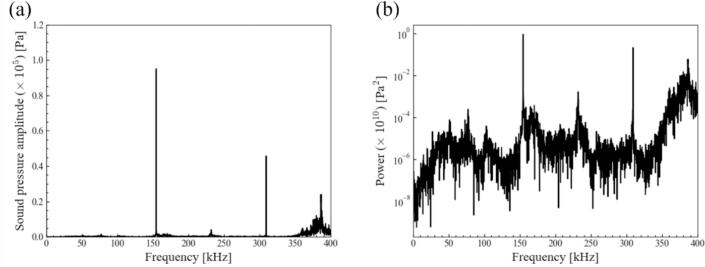
(a) Sound pressure amplitude for each frequency (b) power spectrum for each frequency calculated by a Fast Fourier Transform (FFT) at the ultrasonic power of 69 W.

Fig. 2 shows the waveform of electric voltage measured by the hydrophone. It captured the ultrasonic waves that were propagated from the four transducers and were reflected from the liquid surface. Based on this waveform, the ultrasonic pressure was calculated using a sensitivity data. A fast Fourier transform was performed, and the frequency components in the waveform were calculated. The results are shown in Fig. 3, which indicates the sound pressure amplitude at each frequency and the power spectrum of the sound pressure amplitude at each frequency, respectively. As shown in Fig. 3 (a), it was found that the second harmonic wave at 309 kHz, whose frequency is twice the fundamental frequency of the transducers (154 kHz), and noise appearing at 380 kHz were generated. In this study, these two frequency components are referred to as harmonic waves.

### 2.5 Observation of bubble motion

The bubbles' motion was recorded by a highspeed camera to understand the phenomena. In this observation, two types of bubbles were observed. One is large stable inactive bubbles, and the other is fine active bubbles. The large stable inactive bubbles were observed using a backlight (Leimac, IHMA-214/226RHV). The behavior of the bubbles was observed through a high-speed camera (Photron, FASTCAM AX100), and the frame rate was 1000 or 3000 fps. The fine active bubbles are hard to be observed in this observation. Hence, a laser sheet was used to observe both the fine active and large inactive bubbles. When a laser light was irradiated to a bubble, scattered light was produced from the bubbles. This scattered light was recorded by a high-speed camera. The used laser sheet is the same as that used in the PIV measurement. The frame rate of high-speed camera was set to 1000 fps.

### 2.6 Dissolved oxygen (DO) measurement

Ultrasonic irradiation into an aqueous solution reduces the amount of dissolved oxygen, and this phenomenon is called ultrasonic degassing. In this study, reduction in the dissolved oxygen concentration by the ultrasonic degassing was measured using a DO-meter (HORIBA, D-200–2) to quantify the influence of dissolved gas on quenching. Ultrasound was irradiated into 400 mL pure water for 2.5 min, and the DO was measured every 30 s. The time variation of DO is assumed as a first order reaction. Under this assumption, the degassing rate was evaluated by degassing rate constant *k* [s^−1^] as follows:

(4) $$\frac{dC}{dt}=-kC$$

**Fig. 4:**
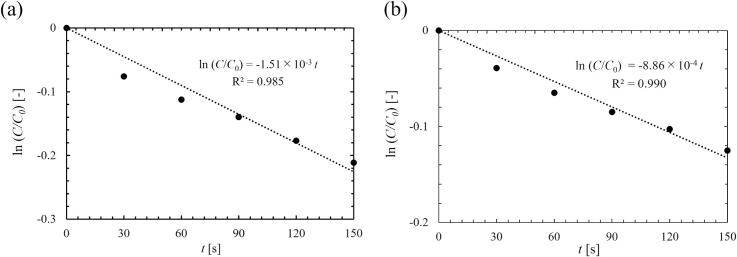
Calculation of degassing rate constant by approximation using Eq. (5) at the ultrasonic power of (a) 33 W, and (b) 84 W.

This equation can be transformed into

(5) $$\ln\frac{C}{C_{0}}=-kt$$

where *C* [mg/L] is the concentration of DO, *C*_0_ is the initial concentration of DO, *t* is time [s]. By using the time variation of dissolved oxygen concentration, the degassing rate constant can be calculated. The example is shown in Fig. 4. Fig. 4 shows a time variation of DO. From the slope shown in Fig. 4, the degassing rate constant was calculated.

## 3 Numerical analysis

In addition to the experiment, we also conducted numerical simulations to understand the experimental results. As explained later, the sound pressure superposition becomes worse near the quenching event. To elucidate the influence of worse superposition on the bubble oscillations, the bubble motion and temperature in the bubble were simulated using the following Keller-Miksis equation [2,44] described as

(6) $$\left(1-\frac{\dot{R}}{c_{\infty }}\right)R\ddot{R}+\frac{3}{2}{\dot{R}}^{2}\left(1-\frac{\dot{R}}{c_{\infty }}\right)=\frac{1}{\rho_{L,\infty }}\left(1+\frac{\dot{R}}{c_{\infty }}\right)\left[p_{B}-p_{s}\left(t\right)-p_{0}\right]+\frac{R}{c_{\infty }\rho_{L,\infty }}\frac{dp_{B}}{\mathrm{dt}}$$

where *R* is the bubble radius, *c*_∞_ is the speed of sound, $\rho_{L,\infty }$ is the liquid density, *p*_B_ is the liquid pressure at the bubble wall, *p*_s_ is the given sound pressure, *p*_0_ is the atmosphere pressure, and *t* is time. The liquid pressure at the bubble wall, *p*_B_ is modeled as

(7) $$p_{B}=p_{g}+p_{v}-\frac{2\sigma }{R}-\frac{4\mu \dot{R}}{R}$$

where *p*_g_ is the pressure inside the bubble, *p*_v_ is the vapor pressure, σ is the surface tension, μ is the dynamic viscosity. The thermodynamic relationship derives the following model for *p*_g_ as

(8) $$p_{g}=\left(p_{0}+\frac{2\sigma }{R_{0}}-p_{v}\right){\left(\frac{R_{0}}{R}\right)}^{3\gamma }$$

where γ is the specific heat ratio. The ultrasonic pressure is modeled as

(9) $$p_{s}=\alpha_{1}P\sin\left(-\omega_{1}t\right)+\alpha_{2}P\sin\left(-\omega_{2}t\right)$$

where *P* is the pressure amplitude, $\alpha_{1}$, $\alpha_{2}$ are the ratio of fundamental and harmonic waves, $\omega_{1}$ is the angular frequency of fundamental ultrasonic wave, and $\omega_{2}$ is the angular frequency of primary harmonic wave. To maintain the same ultrasonic power with different conditions, the following condition was given.

(10) $$\alpha_{1}^{2}+\alpha_{2}^{2}=1$$

This Keller-Miksis equation assumes that the effect of temperature variation in the bubble on the bubble motion is neglected, the bubble shape is always spherical, and the bubble–bubble interaction is neglected. This assumption overestimates the bubble oscillation amplitude in terms of bubble–bubble interaction [45]. In this study, the non-spherical deformation is considered as unstable bubble, which is not evaluated as mentioned later.

To prevent the calculation divergence under the condition for the high-pressure amplitude, the bubble wall velocity is limited at the sound speed at the bubble wall [24,46], *c*_L,B_, which is described as

(11) $$c_{L,B}=\sqrt{\frac{7.15\left(p_{B}+B\right)}{\rho_{L,i}}}$$

where *B* is the pressure parameter, $\rho_{L,i}$ is the liquid density at the bubble wall.

In addition to the bubble motion, the temperature in the bubble was also calculated. The temperature in the bubble, *T* is simply calculated by thermodynamic relationship as

(12) $$T=T_{0}{\left(\frac{R_{0}}{R}\right)}^{\gamma -1}$$

It is to be noted that this calculation roughly estimates the temperature in the bubble [47] although it cannot estimate the temperature accurately because the temperature distribution around the bubble is not considered in this model.

In addition to the bubble motion, the bubble shape stability was numerically investigated to evaluate the effect of harmonic waves on the bubble equilibrium radius. The numerical methods are totally the same as those used in our previous study [48,49]. In this method, the dynamic equation for the bubble shape distortion was solved in addition to the Keller-Miksis equation [50,51]. The solved dynamic equation for the distortion amplitude is described as

(13) $${\ddot{a}}_{n}+B_{n}\left(t\right){\dot{a}}_{n}-A_{n}\left(t\right)a_{n}=0$$

where *A*_n_ and *B*_n_ are model parameters, *a*_n_ is a distortion amplitude, and subscript *n* is degree of spherical harmonics. The parameters, *A*_n_ and *B*_n_ are modeled as

(14) $$A_{n}\left(t\right)=\left(n-1\right)\frac{\ddot{R}}{R}-\frac{\beta_{n}\sigma }{\rho R^{3}}-\frac{2\nu \dot{R}}{R^{3}}\left[\left(n-1\right)\left(n+2\right)+2n\left(n+2\right)\left(n-1\right)\frac{\delta }{R}\right]$$

(15) $$B_{n}\left(t\right)=3\frac{\dot{R}}{R}+\frac{2\nu }{R^{2}}\left[\left(n+2\right)\left(2n+1\right)-2n{\left(n+2\right)}^{2}\frac{\delta }{R}\right]$$

where δ is the thickness of velocity boundary layer, and the $\beta_{n}$ is described as

(16) $$\beta_{n}=\left(n-1\right)\left(n+1\right)\left(n+2\right)$$

After solving these equations, the growth of distortion amplitude was evaluated in an ultrasonic cycle. The bubble shape instability was evaluated by the maximal eigenvalue of the Floquet transition matrix [48]. The shape instability was evaluated for *n* = 2 because the instability with *n* = 2 is the most unstable mode. By calculating the stable condition, the equilibrium bubble size was estimated in this study.

The parameters used in this simulation are given in Table 1. The above equations were solved by 4th order Runge-Kutta method. The used time increment is 5.0 × 10^-4^ ns. The other detailed numerical methods and the validation of simulation program refer to our previous studies [48,49].

**Table 1:** Parameters used in this simulation.

Properties	Value	Unit
Liquid density, *ρ*_L,∞_	9.97 × 10^2^	kgm^−3^
External pressure, *p*_0_	1.00 × 10^5^	Pa
Liquid sound velocity, *c*_∞_	1.50 × 10^3^	ms^−1^
Liquid kinematic viscosity, ν	8.92 × 10^-7^	m^2^s^−1^
Surface tension, *σ*	7.20 × 10^-2^	Nm^−1^
Ratio of specific heat, *γ*	1.40	−
Sound pressure amplitude, *P*	5.00–40.0 × 10^4^	Pa
Equilibrium bubble radius, *R*_0_	0.0800–3.20 × 10^-6^	m
Fundamental angular frequency, *ω*_1_	155,000 × 2π	rads^−1^
Second harmonic angular frequency, *ω*_2_	310,000 × 2π	rads^−1^
Ratio of fundamental wave, *a*_1_	0.7, 1.0	−

## 4 Results and discussion

### 4.1 Chemical reaction rate measured by KI dosimetry

**Fig. 5:**
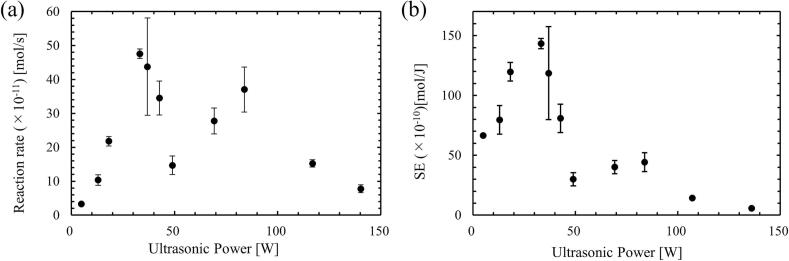
Influence of ultrasonic power on (a) reaction rate and (b) SE.

Fig. 5 shows the influence of ultrasonic power on the reaction rate, *k*_I3_ and on the SE. In Fig. 5(a), the reaction rate, *k*_I3_ increased at low power, reaching the maximum value at the ultrasonic power of 33 W, which is defined as quenching condition. When the ultrasonic power further increased, the reaction rate, *k*_I3_ decreased drastically after the quenching condition. Furthermore, *k*_I3_ increased again above the ultrasonic power of 49 W, while it decreased again at much higher ultrasonic power. The SE, as shown in Fig. 5(b) also showed similar tendency. However, since it is difficult to understand the detailed mechanism of quenching occurrence only from these results, the phenomena occurring in the ultrasonic bath were investigated from the other experiments.

### 4.2 Chemical reaction zones observed by SCL

**Fig. 6:**
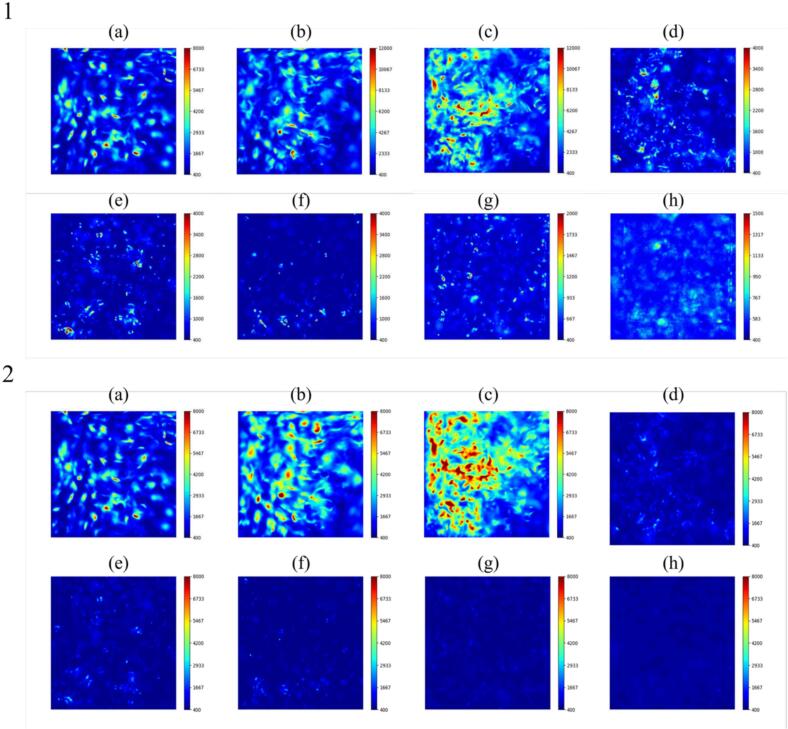
Distribution of sono-chemiluminescence (SCL) intensity and its reaction zone in the ultrasonic bath taken from a window frame (50 mm × 50 mm) with the ultrasonic power of (a) 18 W, (b) 33 W, (c) 43 W, (d) 49 W, (e) 69 W, (f) 84 W, (g) 117 W, and (h) 136 W. The color scales represent the intensity of SCL. 1. The color scale is varied for its visibility and 2. The color scale is same for all powers.

Fig. 6 shows the distribution of instantaneous SCL intensity taken by the EMCCD camera. The color scale in this figure was changed depending on the power so that the reaction zone could be visualized for all powers for Fig. 6.1. In Fig. 6(a)–(c), the SCL intensity was high, and the high-reaction zone spread throughout the vessel, while in Fig. 6(d) – (f), the SCL intensity decreased, and the high-reaction zone was localized. In Fig. 6(g) and (h), the SCL intensity was small, and the reaction zone spread broad. Thus, it is suggested that the pattern of reaction zone formation could be divided into three regions: (a)-(c) for region 1, (d)-(f) for region 2, (g) and (h) for region 3. The ultrasonic power of (c) 43 W is considered to be the transition region between region 1 and region 2 because the error bar is large in Fig. 5, and it is thought that the mechanism changes around the ultrasonic power of (c) 43 W.

**Fig. 7:**
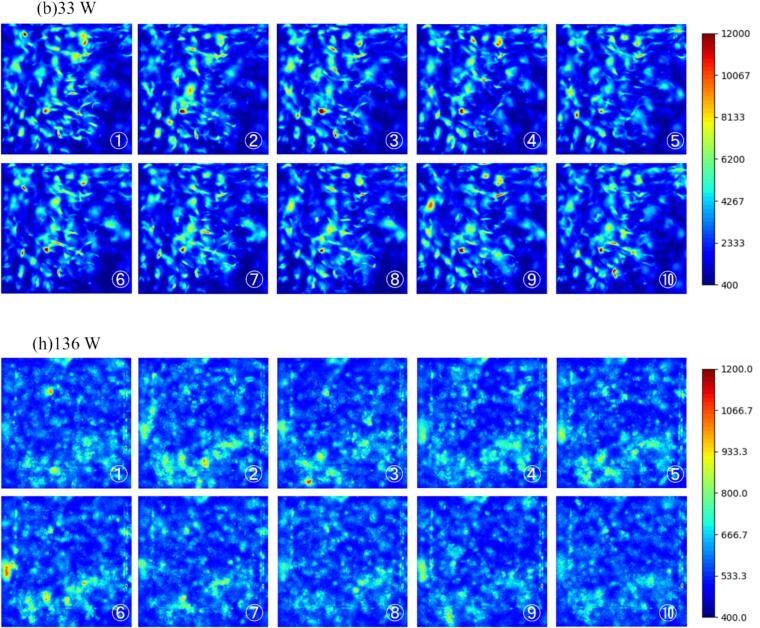
Temporal change of SCL intensity with the ultrasonic power of (b) 33 W, (h) 136 W. The time interval of each photograph is 0.20 s. (Supplemental Video 1).

We also focused on the temporal change of reaction field. The time variations of the reaction field at the ultrasonic power of (b) 33 W and (h) 136 W are shown in Fig. 7. The movie of this SCL result is attached as Supplemental Video 1. It should be noted that the values of the axes in Fig. 7 are different from those in Fig. 6. In Fig. 7(b) at the ultrasonic power of 33 W, the reaction zone did not change temporally very much, and the local high-intense reaction continued to proceed in almost the same zone. On the other hand, as shown in Fig. 7(h) at the ultrasonic power of 136 W, high-intense reaction zones moved significantly. Generally, sonochemical reaction occurs in a zone with high ultrasonic amplitude, suggesting that the temporal changes in the ultrasonic field lead to corresponding changes in the reaction field. Therefore, these results suggest that traveling waves are formed in region 3. It is to be noted that since the propagation speed of traveling waves is so large, it is impossible to capture changes in the reaction field with sufficient time resolution under the present imaging conditions.

### 4.3 Fluid flow velocity obtained by the PIV measurement

**Fig. 8:**
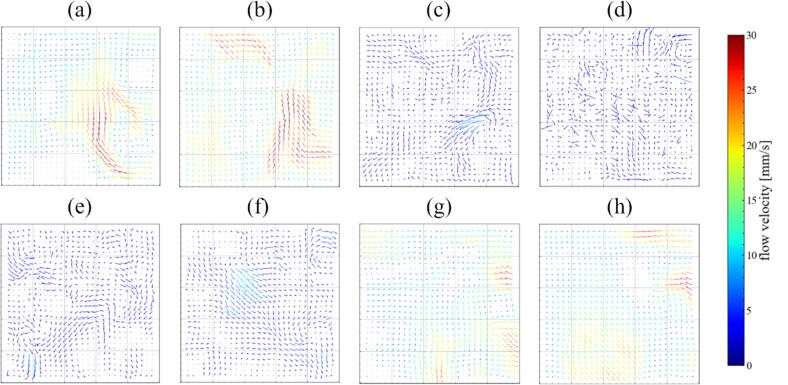
Averaged velocity vectors in the ultrasonic bath with the ultrasonic power of (a) 18 W, (b) 33 W, (c) 43 W, (d) 49 W, (e) 69 W, (f) 84 W, (g) 117 W, and (h) 136 W.

Fig. 8 shows the distribution of the average fluid flow velocity obtained by the PIV measurement. The color of the vectors indicates the magnitude of flow velocity. The fluid flow was not developed in a certain direction, but several local vortices were formed, resulting in a non-uniform direction of the average velocity vector. The average flow velocity was relatively large in Fig. 8(a) and (b), and the maximum velocity was approximately 30 mm/s. As the power increased, the velocity decreased to less than 10 mm/s as in Fig. 8(c)–(f). In Fig. 8(g) and (h), the flow velocity increased again, and the maximum velocity increased to approximately 30 mm/s. Since the fluid is driven by the acoustic streaming, the fluid flow velocity depends on the sound pressure gradient, causing the fluid to move from high-sound pressure to low-sound pressure zone. Therefore, the change in the fluid flow velocity suggests that the sound pressure field is changed with increasing the ultrasonic power and these patterns changes can be divided into three regions similar to the formation of the reaction field in Fig. 7. It is to be noted that the tendency of magnitude in the flow velocity is similar in another cross-sectional plane. Therefore, the same phenomenon occurs throughout the ultrasonic bath.

### 4.4 Sound pressure measured by a hydrophone

**Fig. 9:**
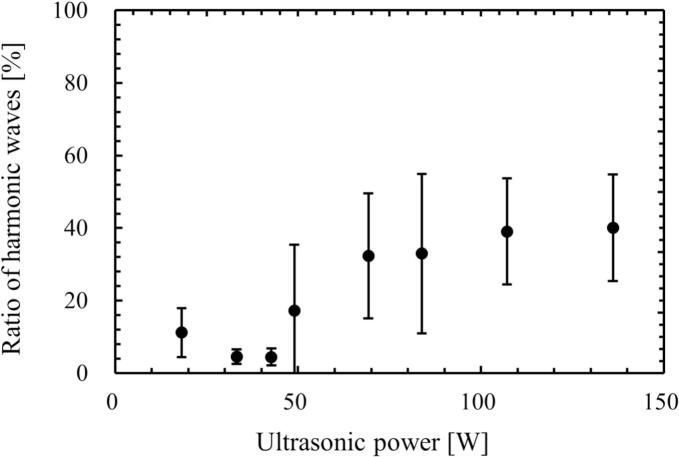
Ratio of harmonic waves measured by the hydrophone. In this result, the harmonic waves consist of second harmonic (309 kHz) and noise (380 kHz).

**Fig. 10:**
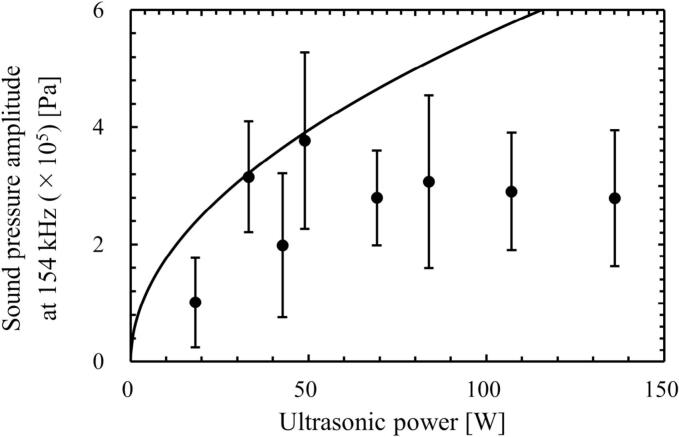
Measured and calculated (solid line) sound pressure amplitudes. The solid line is the theoretical value of sound pressure amplitude calculated by Eq. (17).

The sound pressure at the central location of ultrasonic vessel was measured by the hydrophone, and the time variation of sound pressure was analyzed by the FFT. Fig. 9, Fig. 10 show the relationship between the ultrasonic power and the ratio of harmonic components in the total sound pressure, and the sound pressure amplitude at the fundamental frequency (154 kHz), respectively. The solid line in Fig. 10 is the theoretical value of sound pressure amplitude calculated by Eq. (17), which represents the sound pressure amplitude when the ultrasound waves irradiated from the four transducers are superposed linearly.

(17) $$P=4\sqrt{\frac{\rho_{L}cP_{US}}{A}}$$

where *A* is the irradiation area. The derivation of this equation is described in Appendix A. In region 1 (*P*_US_ < 49 W) in Fig. 9, the ratio of harmonic waves was small, and the measured sound wave was not largely deviated from a sinusoidal wave. However, the ratio of harmonic waves increased at the ultrasonic power more than (d) 49 W. At the same time, the difference between the measured and the theoretical pressure amplitudes increased as the ultrasonic power increased. Therefore, it is found that the superposition of sound waves deteriorated in regions 2 and 3.

**Fig. 11:**
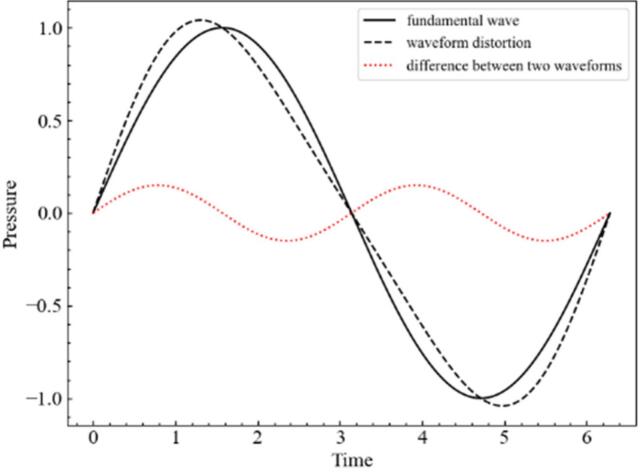
Mechanism of second harmonic wave (309 kHz) generation. The second harmonic wave (red dotted line) is generated by the difference between the linear fundamental wave (solid line) and nonlinear waveform distortion (black dotted line).

Deterioration in the superposition of sound waves is thought to be due to the increased nonlinearity of sound waves. The harmonic wave generation is thought to be mainly caused by (1) nonlinear propagation of ultrasound due to waveform distortion that is caused by cavitation and local temperature variation [52,53], (2) nonspherical oscillation of bubbles (e.g. Guedra *et al.* [54]), and (3) chaotic motion and bubble interaction during bubble oscillations (e.g. Lauterborn and Kurz [55], and Yasui [56]). As shown in Fig. 9, the ratio of harmonic waves was very large especially in regions 2 and 3. Hence, we considered that the mechanisms (2) and (3) are difficult to cause this high ratio of harmonic waves because these two mechanisms are originated from the acoustic radiation from cavitation bubbles. The mechanism (1) of the second harmonic generation is illustrated in Fig. 11. The second harmonic wave (red dot line) is generated by the difference between the linear fundamental wave (solid line) and the wave distortion (black dot line), which is the increased nonlinearity of ultrasound. This reduces the sound pressure amplitude and thereby suppresses the bubble oscillation amplitude because the sound waves are out of phase with each other.

### 4.5 Bubbles motion observed by a high-speed camera

**Fig. 12:**
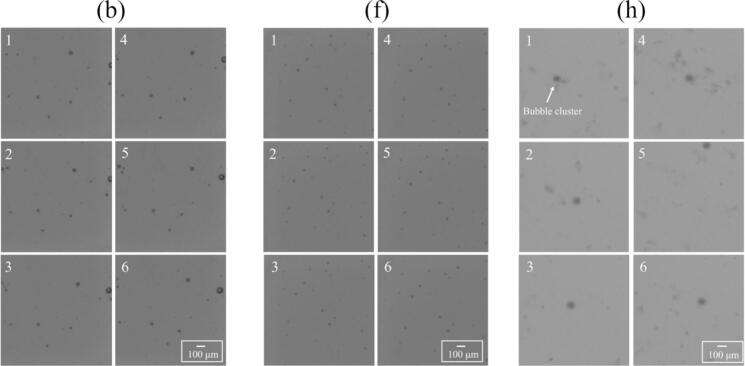
Direct observation of bubbles taken by a highspeed camera with the ultrasonic power of (b) 33 W, (f) 84 W, and (h) 136 W. The time interval of each picture was 0.06 sec. (Supplemental Video2).

Fig. 12 shows the bubbles motion through direct observation using a highspeed camera during ultrasonic irradiation at the ultrasonic powers of (b) 33 W, (f) 84 W, and (h) 136 W for regions 1, 2, and 3, respectively. It should be noted that these bubbles seen in these photographs are large bubbles that are easily observed, and these bubbles are not cavitation bubbles that contribute to the reaction. The position of bubbles did not change with time at the ultrasonic power of (b) 33 W for region 1 and (f) 84 W for region 2. The bubbles are generally trapped at node locations. Therefore, these results indicate that standing waves were formed in regions 1 and 2. On the other hand, at the ultrasonic power of (h) 136 W for region 3, large objects which are considered to be bubble clusters consisting of several bubbles were observed. These bubbles moved around throughout the vessel, suggesting that traveling waves were formed in region 3. We also evaluated the bubbles in these figures by image analysis. The velocity of bubble motion is approximately 5 mm/s, and the central location of bubble did not change very much for the ultrasonic powers of 33 and 84 W. Meanwhile, the velocity of large bubble cluster for 136 W is approximately 100 mm/s, and the motion direction is randomly changed. The movie of this bubble motion is attached in Supplemental Video 2.

**Fig. 13:**
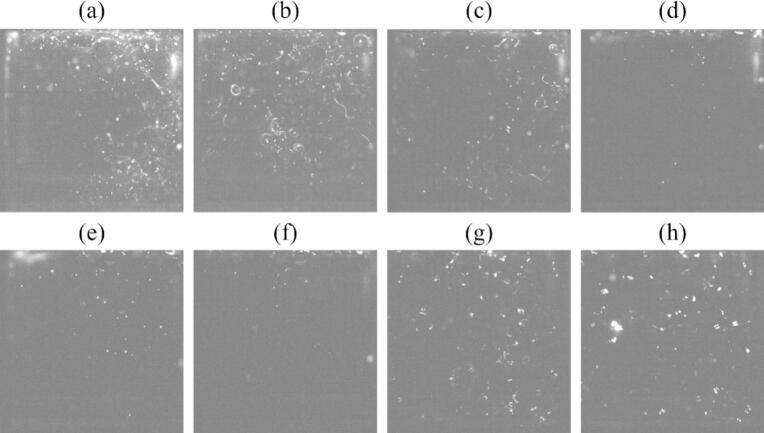
Snapshots of bubbles visualized by the green laser sheet in the ultrasonic bath with the ultrasonic power of (a) 18 W, (b) 33 W, (c) 43 W, (d) 49 W, (e) 69 W, (f) 84 W, (g) 117 W, and (h) 136 W.

**Fig. 14:**
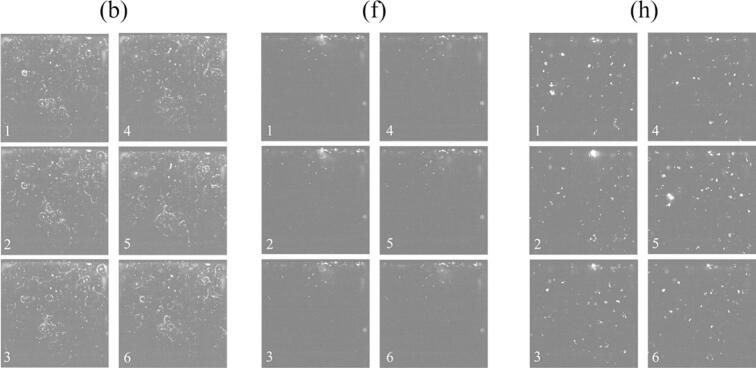
Time variation of bubbles motion and *streamers* formation visualized by green laser in sheet form: with the ultrasonic power of (b)33 W, (f)84 W, (h)136 W. (Supplemental Video 3).

We also observed the bubble motion in another way. A green sheet laser was irradiated to the bubbles, and the scattered light from the bubbles was observed. In this experiment, we could observe two types of bubbles: i. one being relatively large, which is larger than the resonant radius and inactive, and ii. the other being small, which is smaller than the resonant radius and active. The small bubbles form *streamer*, which is a characteristic bubble arrangement, and each bubble moves in a certain direction [32,33,57,58]. Fig. 13 shows the snapshots of observed bubbles with different ultrasonic powers. The number of generated bubbles and the behavior of bubbles showed three different patterns with different regions. Fig. 14 shows the time variation of bubble motion at the ultrasonic power of (b) 33 W, (f) 84 W, and (h) 136 W for regions 1, 2, and 3, respectively. The movie of this bubble motion is attached in Supplemental Video 3. At the ultrasonic power of (b) 33 W for region 1, it can be seen that a lot of bubbles were generated, and *streamers* were formed. In this experiment, the trajectory of the bubble movement formed a circle. Therefore, we named this phenomenon *rotating streamers*. On the other hand, at the ultrasonic power of (f) 84 W for region 2, the number of bubbles and *streamers* obviously decreased. Finally, at the ultrasonic power of (h) 136 W for region 3, the number of bubbles increased again. In addition, the size of bubbles was larger than that in regions 1 and 2, and the shape of bubbles was distorted instead of a regular circle. This result is another evidence of formation of bubble clusters, which move around violently as shown in Fig. 12 (h).

### 4.6 Degassing rate measured by a DO meter

**Fig. 15:**
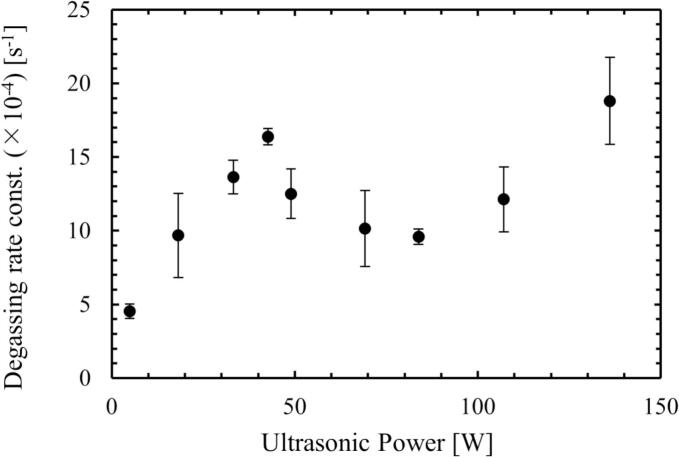
Relationship between the ultrasonic power and the degassing rate constant.

Acoustic bubbles grow to a certain size during repeating expansion and contraction. During this growth, the dissolved gas is transported into the bubble due to rectified diffusion [3,59]. Some bubbles move toward a specific location, and they coalesce to form larger bubbles. Finally, the bubbles rise to the free surface, then degassing occurs. Some bubbles are collapsed, and chemical reactions proceed. Some of the collapsed bubbles become tiny bubbles again, so the gas species return to the aqueous solution. In this subsection, we investigated the influence of ultrasonic power on the degassing rate. Fig. 15 shows the relationship between the ultrasonic power and degassing rate constant. The degassing rate constant increased with increasing the ultrasonic power at the ultrasonic power of (a)-(c) in region 1. In region 1, although the bubbles formed *streamers* and stayed in the solution for a long time, the degassing rate constant in region 1 was sufficiently large, indicating that the degassing is effectively promoted. On the other hand, the degassing rate constant decreased at the ultrasonic power of (d)-(f) for region 2 and finally increased at the ultrasonic power of (g) and (h) for region 3. In region 2, the bubble generation rate decreased, and the number of formed bubbles was small as shown in Fig. 13, Fig. 14. In region 3, the degassing rate increased due to increasing the number of bubbles generated, that formed bubbles clusters.

### 4.7 Discussion about the formation mechanism of quenching

**Fig. 16:**
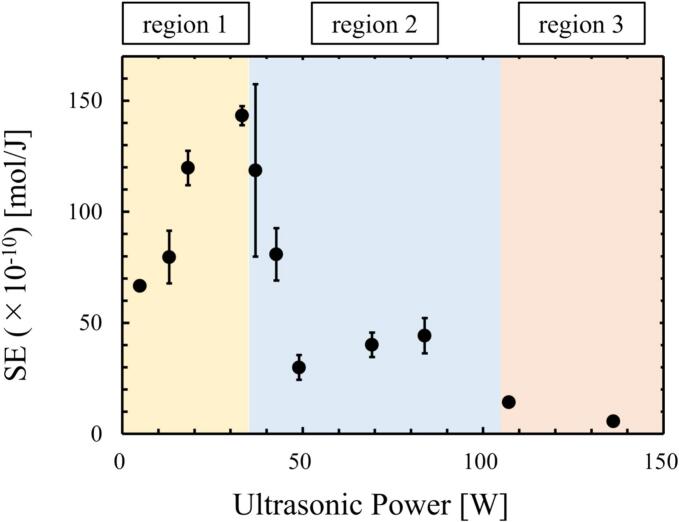
Three regions identified by this experiment.

The summary of the experimental results is shown in Fig. 16 and Table 2. The phenomena can be divided into three regions in terms of the mechanism as explained above. When the phenomena occurring during the ultrasonic irradiation are changed from region 1 to region 2, the superposition of sound waves deteriorated, limiting the reaction zone and thereby the reaction rate decreased. On the other hand, when the phenomena are changed from region 2 to region 3, the reaction rate was further decreased due to the formation of bubble clusters and traveling ultrasonic waves. The detailed mechanism changes are described below.

**Table 2:** Summary of experimental results in three regions.

	Region 1	Region 2	Region 3
Ultrasonic power, *P*_US_	<35 W	35–100 W	100 W<
Chemical reaction	High	Low	Low
Velocity of acoustic streaming	High 20–30 mm/s	Low < 10 mm/s	High 20–30 mm/s
Wave superposition	High	Low	Low
Harmonic ratio	<10 %	20–40 %	20–40 %
Pressure field	Standing wave	Standing wave	Traveling wave
Number of bubbles	Many	Few	Many (Bubble cluster)
Degassing rate	High	Low	High

**Fig. 17:**
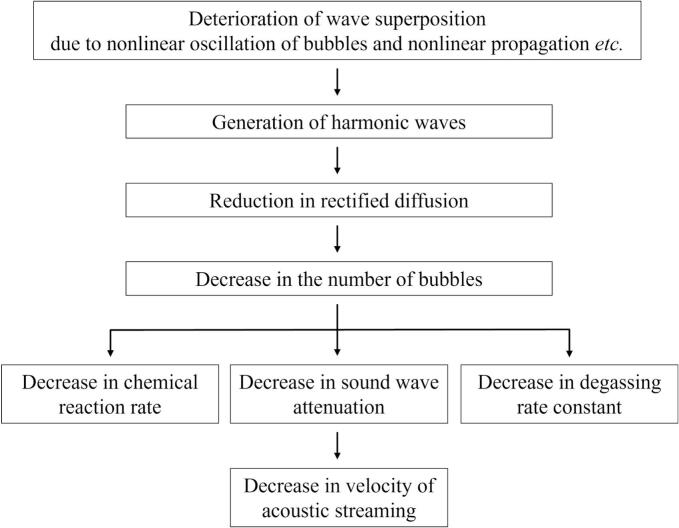
Schematic diagram of mechanism changes from region 1 to region 2.

Fig. 17 shows the schematic diagram of mechanism changes from region 1 to region 2. In region 1, cavitation bubbles oscillate significantly, then chemical reactions with large reaction rate proceed throughout the vessel because standing waves are formed, and the superposition of ultrasound is good. As the results, the amount of nucleated bubble increases, and *streamers* and coalesced large bubbles are formed. Therefore, the larger reaction rate with increase of the ultrasonic power in region 1 is originated from the increase in the number of oscillating bubbles. However, the superposition of sound waves deteriorates, and harmonic waves are generated due to the increased nonlinearity of the ultrasonic waves in region 2. As discussed later from the numerical results, the harmonic waves suppress bubble expansions that reduces the rectified diffusion. As the results, the number of produced bubbles decreases. As shown in Fig. 17, the decrease in the number of produced bubbles causes the following three phenomena: i. decrease in chemical reaction rate, ii. decrease in the sound wave attenuation, and iii. decrease in the degassing rate. The first phenomenon is the decrease in chemical reaction rate. Since chemical reactions proceed in the cavitation bubble, small number of cavitation bubbles decreases the whole reaction rate in the ultrasonic bath. The second phenomenon is decrease in the sound wave attenuation. When a kHz ultrasound is used in the presence of cavitation bubbles, the ultrasonic energy is mainly converted to bubble motion and the influence of viscous dissipation is very small. Therefore, the ultrasonic waves are attenuated mainly by cavitation bubbles. Thus, the decrease in the number of cavitation bubbles reduces the absorption of ultrasonic energy, which is the driving force of acoustic streaming, resulting in the decrease in acoustic streaming velocity as shown in Fig. 8. In the previous study [37], the fluid velocity of acoustic streaming is drastically reduced in a highly degassed water. The result of this previous study corresponds to the above discussion. The third phenomenon is decrease in the degassing rate. As the number of cavitation bubbles decreases, the number of coalesced large bubbles decreases and the floating large bubbles on the free surface also decrease.

**Fig. 18:**
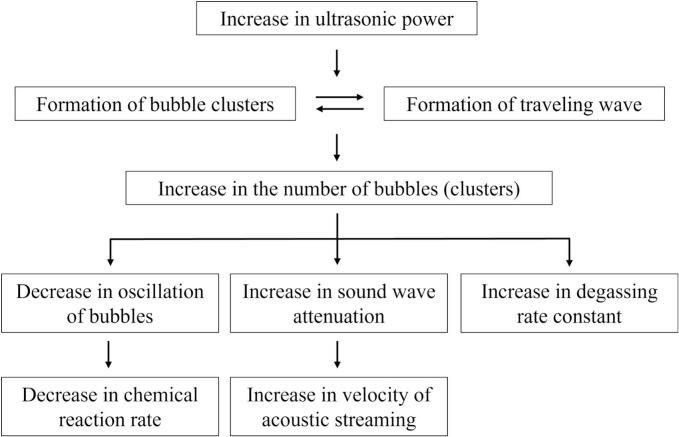
Schematic diagram of mechanism changes from region 2 to region 3.

Fig. 18 shows the schematic diagram of mechanism changes from region 2 to region 3. In region 3, the secondary Bjerknes force becomes large with increasing ultrasonic power. As a result, the attraction force between bubbles acts strongly, forming bubble clusters. Thus, the formation of bubble clusters changes the sound waves propagation and forms traveling waves in region 3. The bubble clusters are not trapped and continue to move in the direction of sound waves with traveling waves. Chemical reaction rate decreases due to small amplitude of bubble oscillations in the bubble clusters and traveling waves, as shown in Fig. 6(h). In addition, the flow velocity of acoustic streaming increases because of increased attenuation of ultrasound, which is caused by the increase in the number of generated bubbles. Moreover, the increase in the degassing rate is caused by an increase in the number of formed bubbles, and the generated bubbles move to the free surface in traveling waves.

Although we have discussed the experimental results and the mechanism, there are unclear points about the way how the bubble behavior and chemical reaction rate change due to harmonic waves. Numerical simulation can clarify them. The numerical simulation was conducted to clarify the influence of harmonic waves on the equilibrium bubble radius, bubble oscillation, and the temperature and chemical reaction rate in the bubble.

The influence of harmonic waves on the equilibrium radius was evaluated by the shape stability of acoustic cavitation under an ultrasound with a harmonic wave. The influence of harmonic waves on the maximum temperature in the bubble in an ultrasonic cycle was evaluated by solving the Keller-Miksis equation under the stable zone, which was calculated by the stability analysis. Finally, the influence of harmonic waves on the bubble expansion and growth was evaluated by solving the Keller-Miksis equation for small bubbles.

**Fig. 19:**
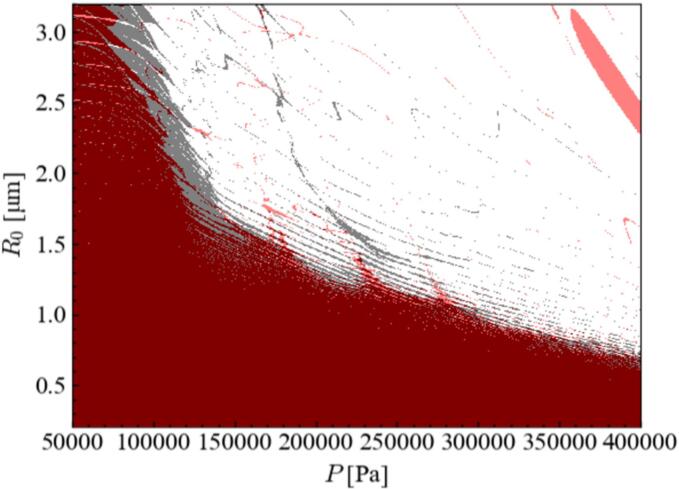
Stability diagram for bubble shape instability with and without harmonic waves: The black color is stable zone for *α*_1_ = 1.0, and the red color is stable zone for *α*_1_ = 0.7.

Fig. 19 shows the stability diagram with or without harmonic waves. In this figure, the black color is stable zone for *α*_1_ = 1.0, and the red color is stable zone for *α*_1_ = 0.7. As can be seen from this figure, the stable zone is slightly changed due to the harmonic waves although this discrepancy is very small especially for the condition with the high-pressure amplitude where the chemical reactions proceed in the cavitation bubbles. From these results, we considered that the influence of harmonic waves on the equilibrium bubble radius is negligibly small. Therefore, the equilibrium bubble radius is not changed due to the harmonic waves near the quenching condition and the quenching of chemical reaction is not caused by the change in the equilibrium bubble radius.

**Fig. 20:**
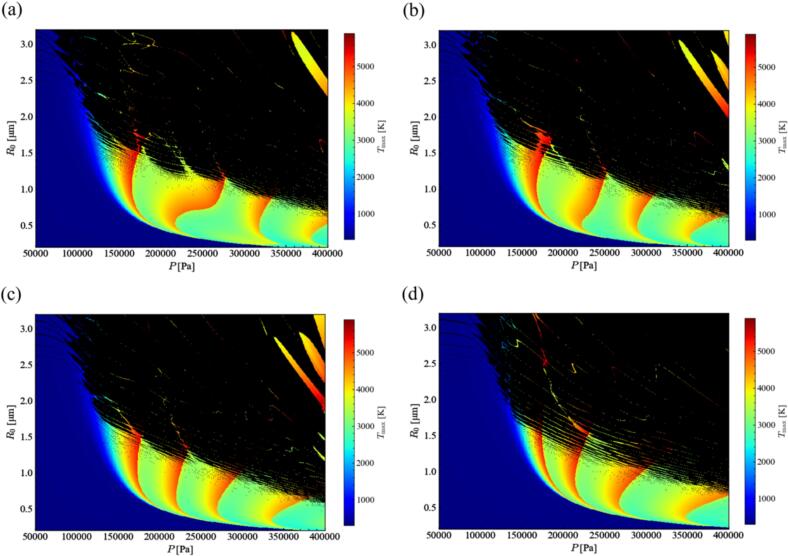
Distribution of the maximum temperature in the bubble in an ultrasonic cycle with different ratio of fundamental frequency, *α*_1_: (a) 0.7, (b) 0.8, (c) 0.9, and (d) 1.0. The black zone indicates the unstable zone, where the stable bubble cannot exist.

Fig. 20 shows the distribution of the maximum temperature in the bubble in an ultrasonic cycle with different ratio of fundamental frequency. In these figures, the black part indicates the unstable zone, where the bubble is easily fragmented [60]. The bubble inner temperature becomes high in the four stripped zones in both cases with and without harmonic waves. And the whole distribution is similar except for the stripped zones with high-pressure amplitude. From these results, we considered that the maximum temperature in the bubble is not changed largely due to the harmonic waves. Therefore, the influence of harmonic waves on the bubble inner temperature cannot be the main factor to change the chemical reaction rate.

**Fig. 21:**
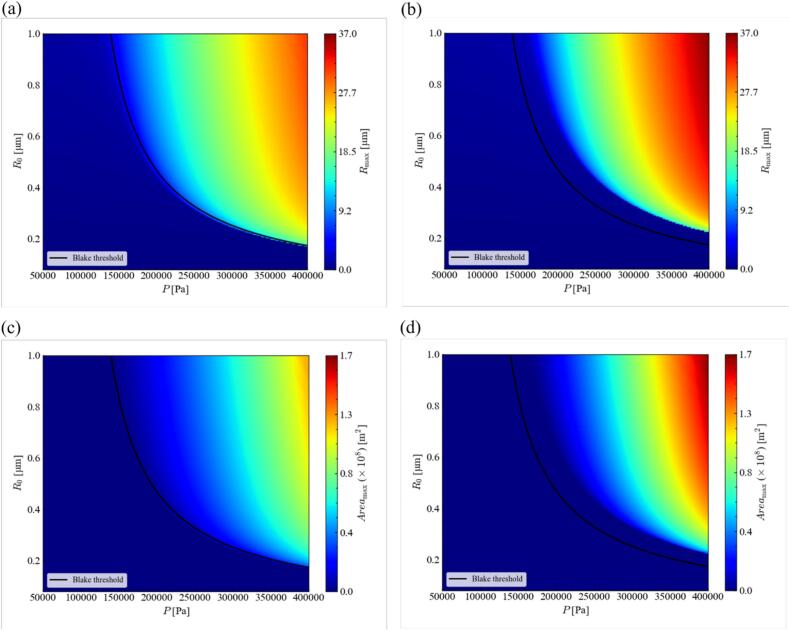
Distributions of (a), (b) maximum bubble radius and (c), (d) maximum bubble interfacial area with and without harmonic waves; *α*_1_ = (a), (c) 0.7, and (b), (d) 1.0. The black line indicates the Blake threshold.

Finally, we discuss the effect of harmonic waves on the bubble oscillation amplitude. Fig. 21 shows the distribution of the maximum bubble radius and the maximum bubble interfacial area in an ultrasonic cycle with or without harmonic waves. In this figure, the black line indicates the Blake threshold, which is described as

(18) $$A_{\mathrm{Blake}}=p_{0}-p_{v}+\frac{4\sigma }{3}\sqrt{\frac{2\sigma }{3R_{0}^{3}\left(p_{0}+\frac{2\sigma }{R_{0}}-p_{v}\right)}}$$

where *A*_Blake_ is the Blake threshold. The cavitation bubble cannot be generated below this threshold. In these figures, cavitation bubbles cannot be generated in the left part from this threshold. As can be seen clearly from these figures, the harmonic waves weaken the bubble oscillations, and the maximum bubble radius becomes small. Accordingly, the bubble interfacial area decreases largely with harmonic waves. This phenomenon weakens the rectified diffusion [3,61] resulting in the suppression of bubble growth and bubble formation quantity. The dissolved gas specie is transported into the bubble during nonlinear bubble oscillations, which is called rectified diffusion. The rectified diffusion is intensified because the interfacial area of bubble is large and the concentration boundary layer becomes thin when the bubble is expanded. These phenomena are called area affect and shell effect. The surface area in the case without harmonic wave is much larger than that in the case with harmonic waves, the results of which are shown in Fig. 21(c) and (d). Hence, the rectified diffusion decreases largely, and the bubble growth rate decreases drastically with harmonic waves. This result corresponds well to the experimental result of degassing. The generation of harmonic waves suppresses the bubble growth, and it decreases the number of formed bubbles. Finally, the whole chemical reaction rate decreases due to harmonic waves generation.

### 4.8 Comparison with the other experiment and other proposed mechanism

The generation mechanism of quenching is compared with the other previous studies. Initially, the quenching was found, and the mechanism was explained in terms of the bubble cluster formation by Hatanaka *et al*. [34,35]. This mechanism corresponds to the mechanism change from region 2 to region 3 in this study. However, this mechanism is one of the quenching mechanisms proposed in this study. Later, Tuziuti *et al*. [36] found the quenching occurrence shifts to the higher-pressure condition by addition of fine particles. They discussed that the reflection of ultrasound at the free surface is changed due to the accumulation of added particles on the free surface, and the standing wave is easily formed with the particle addition. They also argued that the chemical reaction rate decreases due to the unstable ultrasonic field, which is caused by the attenuation and scattering of ultrasound due to formed cavitation bubble. We totally agreed with this discussion. This discussion corresponds to our experimental results obtained by hydrophone as shown in Fig. 9, Fig. 10. However, the suppression of bubble formation and degassing rate after the first quenching event cannot be explained from this previous study. Lee *et al*. [37] explained that the quenching is caused by the traveling wave component. They found that the chemical reaction rate becomes low with increase in the traveling wave component. This result corresponds to our experimental results of deterioration of sinusoidal ultrasonic wave as shown in Fig. 9, Fig. 10. However, the explanation about the suppression of bubble formation and degassing rate obtained in this study cannot be explained from this previous study [37]. More recently, Asakura and Yasuda [38] investigated the quenching event with different frequencies. In this study, the calorimetry method sufficiently detects the quenching condition, and they discussed the mechanism of quenching formation. They claimed that the number of created cavitation bubbles influences the quenching, and the number of formed bubbles decreased after the quenching event although they did not show the experimental results. They explained this phenomenon in terms of Bjerknes force. However, there is a contradiction in this explanation. If the number of formed bubbles is reduced after the quenching event, the scattering and attenuation of ultrasound due to bubbles should decrease, and the Bjerknes force should decrease, making quenching less likely to occur. There is a contradiction that an increase in the number of formed bubbles causes the standing wave to be deteriorated and thus the number of formed bubbles to decrease. Although the experimental results shown in this study well correspond to the discussion in this previous study [38], we support the mechanism proposed in this study. Firstly, the superposition of ultrasound is deteriorated, and the harmonic waves are formed due to nonlinear propagation of ultrasound, which is mainly caused by the existence of acoustic cavitation. Then, the cavitation nucleation and the bubble expansion due to rectified diffusion are suppressed by the harmonic waves and weakly superposed ultrasound as shown in Fig. 21. As the results, the chemical reaction rate decreases, the degassing rate decreases, and the attenuation of ultrasound decreases, resulting in the smaller velocity of acoustic streaming.

## 5 Conclusion

In this study, the mechanism of quenching was investigated under conditions in which ultrasound was irradiated from four transducers at 154 kHz and the liquid surface was fixed with an acrylic plate. It was found that the mechanism of power-induced quenching can be divided into three regions. The main findings of this study are summarized as follows:

• At low ultrasonic power, standing waves were formed and the ultrasonic pressure amplitude was large because the superposition of ultrasound was good. Therefore, the bubbles oscillated largely, and the reaction rate was high.
• At middle ultrasonic power, nonlinearity of sound waves increased, harmonic waves were generated, and the superposition of ultrasound was deteriorated. The numerical results showed that the expansion of bubbles was suppressed. Accordingly, the rectified diffusion became smaller. As a result, the number of bubbles decreased, and then the sonochemical efficiency and the degassing rate decreased significantly. In addition, the attenuation of ultrasound became small resulting in the decrease of fluid flow velocity due to acoustic streaming.
• At high ultrasonic power, the secondary Bjerknes force acted strongly between bubbles, forming bubble clusters. Thus, traveling waves were formed, and the bubbles moved around with traveling waves. Therefore, bubbles oscillation decreased, and the SE decreased further.

## CRediT authorship contribution statement

**Ryota Aoki:** Writing – original draft, Visualization, Investigation, Formal analysis, Data curation. **Kanji D. Hattori:** Writing – review & editing, Visualization, Validation, Investigation, Formal analysis, Data curation. **Takuya Yamamoto:** Writing – review & editing, Validation, Supervision, Project administration, Methodology, Funding acquisition, Conceptualization.

## Declaration of competing interest

The authors declare the following financial interests/personal relationships which may be considered as potential competing interests: Takuya Yamamoto reports financial support was provided by Japan Science and Technology Agency. If there are other authors, they declare that they have no known competing financial interests or personal relationships that could have appeared to influence the work reported in this paper.

## Appendix

### Appendix A Derivation of maximum pressure amplitude of superposed ultrasound

The maximum pressure amplitude of superposed ultrasound is derived below. The ultrasound was irradiated from three planes in this vessel: one was from the bottom, and the others were from the two sides. We assumed the situation where all the ultrasound is superposed well and the incident wave to the top surface is perfectly reflected. In this condition, the superposed ultrasonic waves in the vertical direction are described as

(A1) $$p_{\mathrm{ys}}=p_{i}+p_{r}$$

where *p*_ys_ is the superposed ultrasound in the vertical direction, *p*_i_ is the incident wave, and *p*_r_, which are represented as

(A2) $$p_{i}=P_{B}\sin\left(-ky+\omega t\right)$$

(A3) $$p_{r}={-P}_{B}\sin\left(ky+\omega t\right)$$

where *P*_B_ is the pressure amplitude at the bottom surface, *k* is the wavelength, *y* is the vertical coordinate, ω is the angular frequency, and *t* is time. By substituting Eqs. (A2), (A3) into Eq. (A1), the following superposed ultrasonic waves can be obtained.

(A4) $$p_{\mathrm{ys}}=P_{B}\left[\sin\left(-ky+\omega t\right)-\sin\left(ky+\omega t\right)\right]=2P_{B}\cos\left(\omega t\right)\sin\left(-ky\right)$$

Next, we consider the ultrasound from the sides. The ultrasound from the sides, *p*_side1_ and *p*_side2_ is described as

(A5) $$p_{side1}=P_{s}\sin\left(kx-\omega t\right)$$

(A6) $$p_{side2}=P_{s}\sin\left(kx+\omega t\right)$$

where *P*_s_ is the pressure amplitude from the side. The vessel width is almost 8 times larger than the wavelength of ultrasound. In this case, the horizontal ultrasound waves are superposed as

(A7) $$p_{\mathrm{xs}}=p_{side1}+p_{side2}=2P_{s}\cos\left(-\omega t\right)\sin\left(kx\right)$$

All the linearly superposed ultrasound is described as

(A8) $${p=p}_{\mathrm{xs}}+p_{\mathrm{ys}}=2P_{B}\cos\left(\omega t\right)\sin\left(-ky\right)+2P_{s}\cos\left(-\omega t\right)\sin\left(kx\right)=\cos\left(\omega t\right)\left[\left(2P_{B}\sin\left(-ky\right)+2P_{S}\sin\left(kx\right)\right)\right]$$

The pressure amplitudes at the bottom and side surfaces are the same in this vessel. Therefore, the pressure is finally transformed into

(A9) $${p=p}_{\mathrm{xs}}+p_{\mathrm{ys}}=2P\cos\left(\omega t\right)\left[\sin\left(-ky\right)+\sin\left(kx\right)\right]$$

The term of [sin(−*ky*) + sin (*kx*)] is ranged from −2 to 2. Therefore, the maximum pressure amplitude of superposed ultrasound in this vessel is 4 *P*.
