# Supplementary figures and images for: Revisit to the mechanism of quenching: Power effects for sonochemical reactions
Source: Ultrason Sonochem. 2025 Jun 6;120:107419. doi: 10.1016/j.ultsonch.2025.107419 (PMC12178931; doi:10.1016/j.ultsonch.2025.107419)

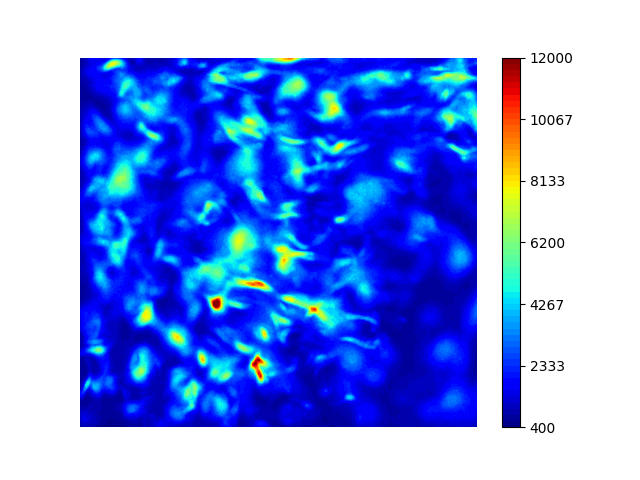

Supplement: Supplementary Video 1b [file mmc7.zip › 50W_time variation.gif]

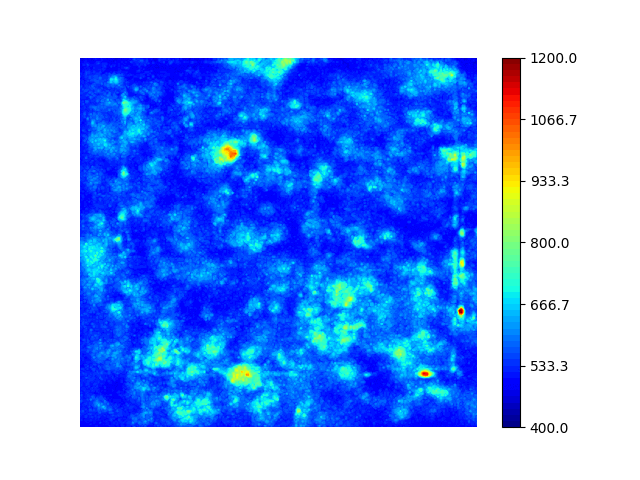

Supplement: Supplementary Video 1h [file mmc1.zip › 250W_time variation.gif]

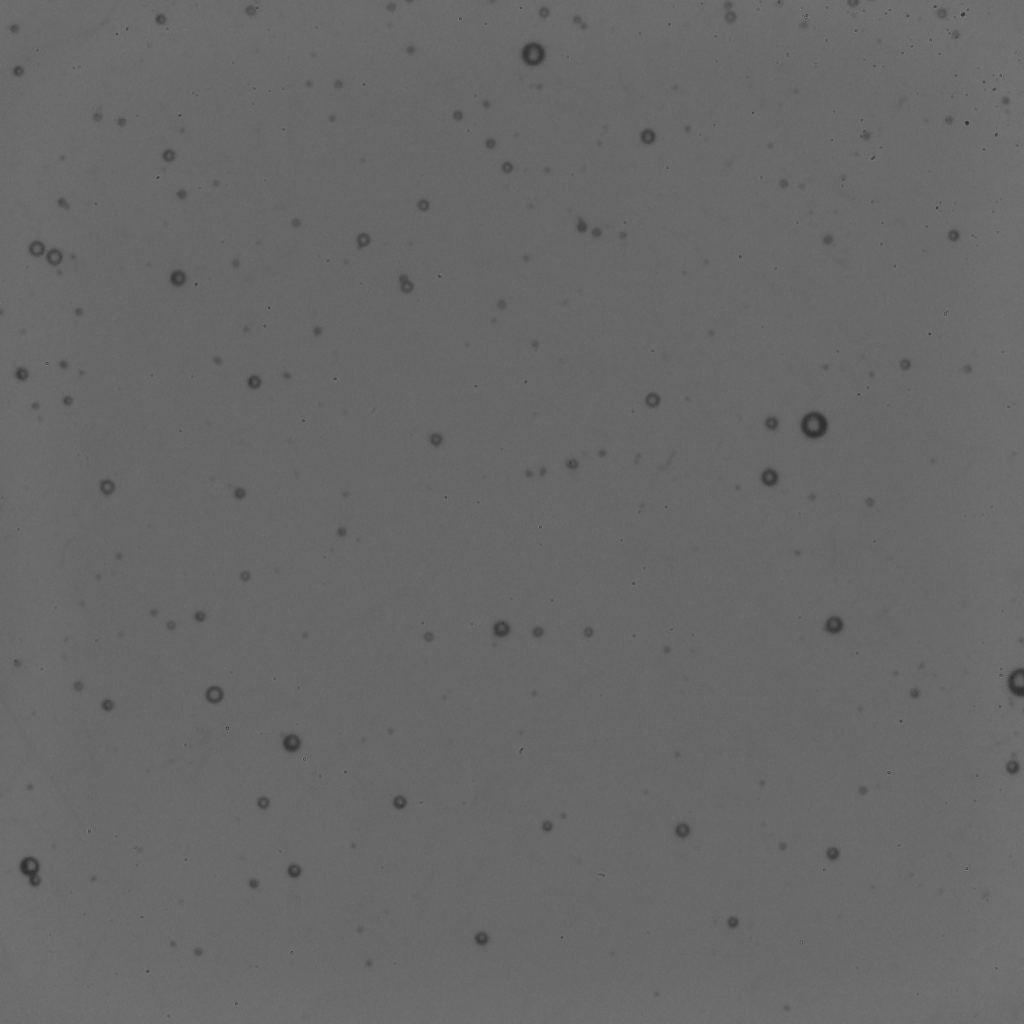

Supplement: Supplementary Video 2b [file mmc8.zip › 33W_direct observation.gif]

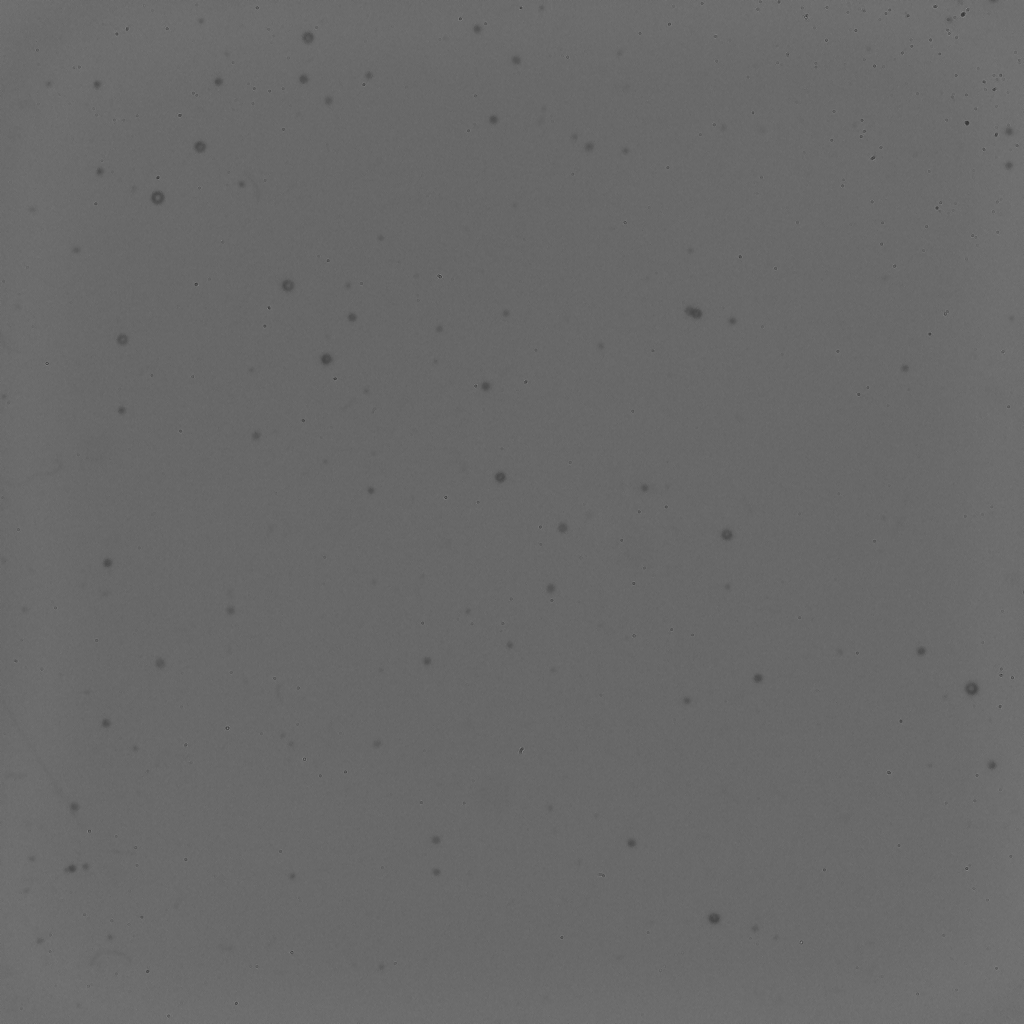

Supplement: Supplementary Video 2f [file mmc5.zip › 84W_direct observation.gif]

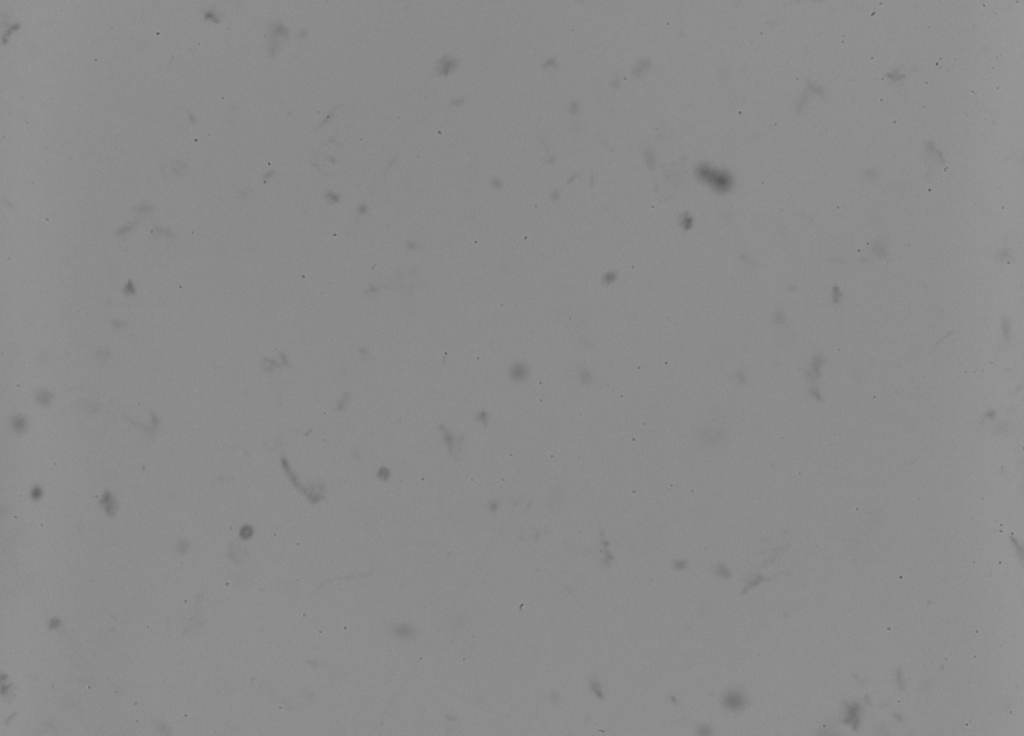

Supplement: Supplementary Video 2h [file mmc3.zip › 136W_direct observation.gif]

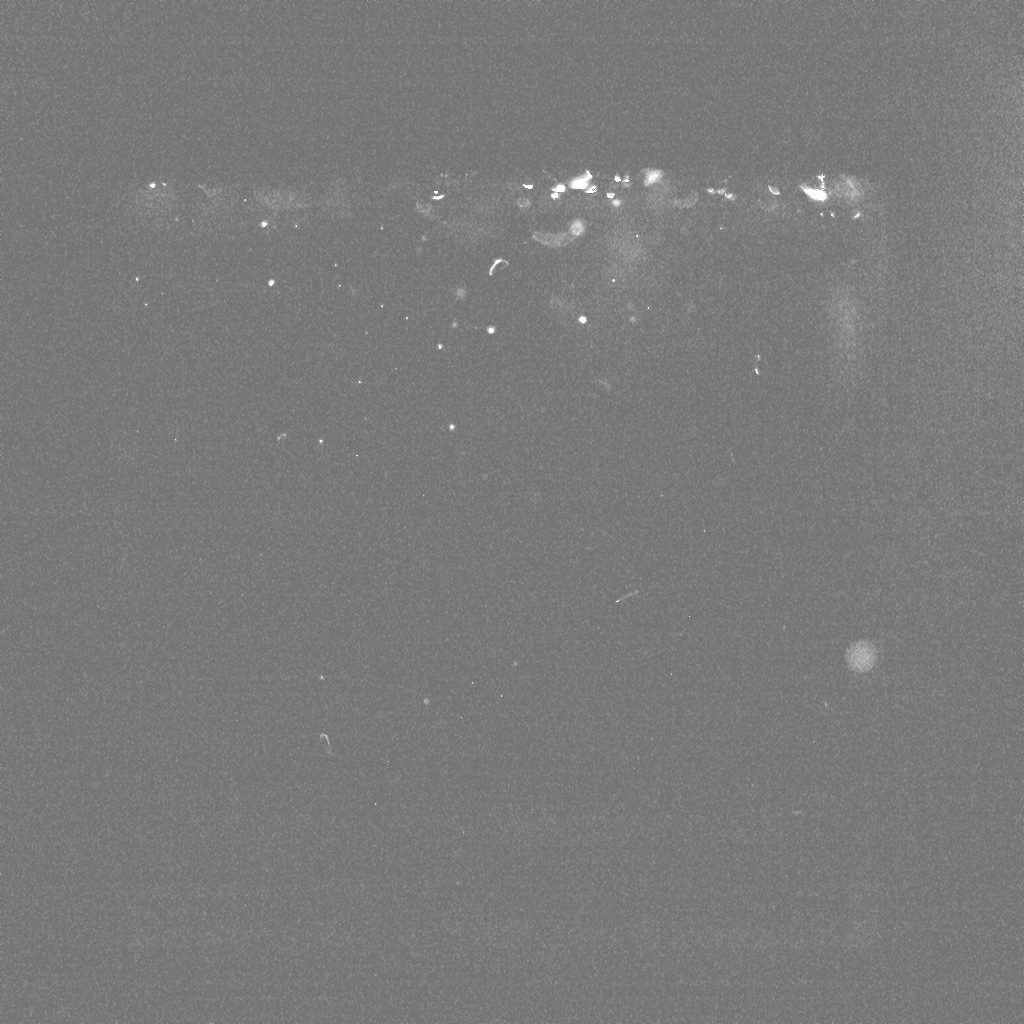

Supplement: Supplementary Video 3f [file mmc4.zip › 84W_green.gif.gif]

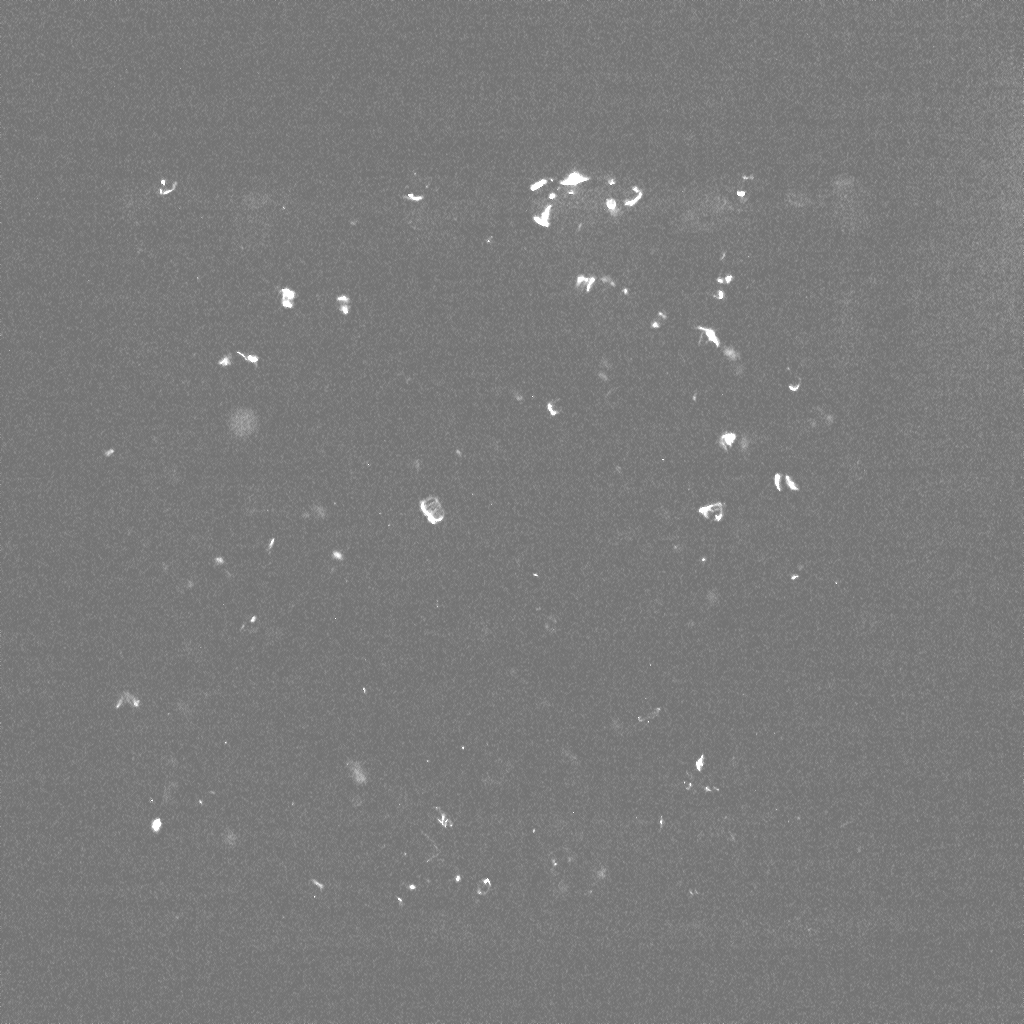

Supplement: Supplementary Video 3h [file mmc2.zip › 136W_green.gif.gif]
